# A Rare Case Report of Extra-adrenal Pheochromocytoma with Normal Blood Pressure: Is that Possible?

**DOI:** 10.7759/cureus.3167

**Published:** 2018-08-20

**Authors:** Ahmed Elkheshen, Masud Billah, Amir Shahbaz, Paria Zarghamravanbakhsh, Usman Nabi, Issac Sachmechi

**Affiliations:** 1 Internal Medicine, The Icahn School of Medicine at Mount Sinai, New York, USA; 2 Internal Medicine, Icahn School of Medicine, Mount Sinai/ Queens Hospital Center, New York, USA; 3 Internal Medicine, Icahn School of Medicine at Mount Sinai/Queens Hospital Center, New York, USA; 4 Endocrinology, Icahn School of Medicine at Mount Sinai Queens Hospital Center, New York, USA; 5 Diagnostic Radiology, Hamad General Hospital, Doha, QAT

**Keywords:** extra-adrenal pheochromocytoma, hypokalemia, hypomagnesemia, hypocalcemia, normotensive

## Abstract

Extra-adrenal pheochromocytoma is uncommon and usually secreting nor-epinephrine. We are presenting a possible case of extra-adrenal pheochromocytoma in a 68-year-old male who was admitted to Queens Hospital Center complaining of shortness of breath for two days. Physical examination was unremarkable except tachycardia. Ventilation/perfusion (V/Q) scan showed the intermediate probability for pulmonary thromboembolism. Computed tomography (CT) chest confirmed the presence of old embolism and showed the 1.1 cm nodule in the left upper lobe. He suddenly collapsed and went into cardio-respiratory failure and attempts to resuscitate were futile. Results for pheochromocytoma workup received after the patient has passed away and it showed elevated levels of 24-hour urine metanephrine, normetanephrine, and Vanillylmandelic acid (VMA). In our patient, CT abdomen did not identify any mass in the adrenal gland or at the bifurcation of the aorta. The extra-adrenal tumor can secrete enough epinephrine to negate the hypertensive effect of norepinephrine. The clinician should be aware of the possibility that tachycardia could be a presenting symptom in pheochromocytoma although the patient is normotensive.

## Introduction

Phaeochromocytomas are catecholamine-producing neuroendocrine tumors originating from chromaffin cells of the adrenal medulla or extra-adrenal paraganglia. Tumors from extra-adrenal chromaffin tissue are titled as extra-adrenal pheochromocytomas or paragangliomas [1]. The term paraganglioma is also labeled for the parasympathetic tissue tumors in the head and neck; these tumors do not secrete catecholamines. Nearly 80–85% of phaeochromocytomas arise from the adrenal medulla, whereas about 15–20% are from the extra-adrenal chromaffin tissue [1, 2]. Extra-adrenal paragangliomas are usually found in the abdomen [3]. The prevalence of phaeochromocytoma in the hypertension outpatient clinic is 0.1–0.6% [4]. Although these tumors are frequently searched for, they are rarely found. The relatively high prevalence of phaeochromocytoma in autopsy studies (about 0.05%) also indicates that many tumors are missed, resulting in premature mortality [5, 6]. Most of the clinical signs and symptoms of phaeochromocytoma are due to the direct actions of secreted catecholamines—hypertension, tachycardia, and feelings of panic or anxiety [7]. We are presenting a possible case of extra-adrenal pheochromocytoma emitting an equal amount of epinephrine and norepinephrine, which might be the leading cause of normal blood pressure in such patients.

## Case presentation

**Figure 1:**
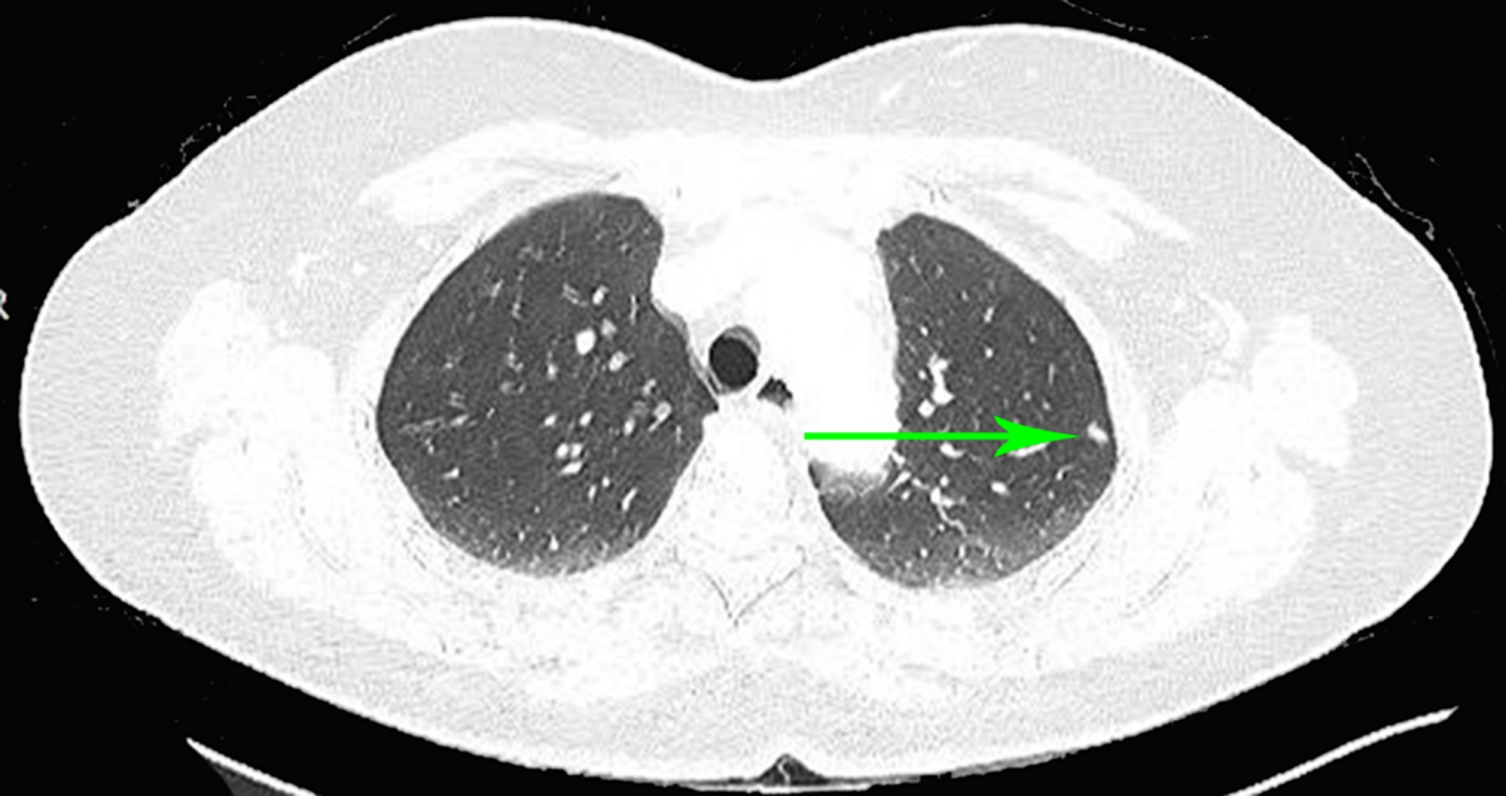
Computed tomography (CT) chest: green arrow pointing at 1.1 cm nodule in the left upper lung lobe.

A 68-year-old male was admitted to Queens Hospital Center with a complaint of shortness of breath for two days duration. The patient had been noticing a decrease in exercise tolerance for a few weeks. Two weeks earlier he was admitted to Queens Hospital with a complaint of chest pain, and acute coronary syndrome was ruled out. On evaluation, the patient had tachycardia with a heart rate of 120–124/min and blood pressure (BP) of 110/80 mm Hg. Lungs were clear to auscultation, and there was no evidence of infection or blood loss. The patient was empirically started on anticoagulation for pulmonary embolism. Computed tomography (CT) chest was not done initially due to elevated creatinine of 1.6, ventilation/perfusion scan (V/Q scan) showed the intermediate probability for pulmonary thromboembolism. The patient was continued on anticoagulation. However, patient tachycardia was persistent, and a blood test showed persistent hypocalcemia 6.8–7.9 mg/dL and hypokalemia 3.2–3.4 mEQ/L with normal thyroid function test. 25-hydroxyvitamin D test was low (6 ng/ml) and intact parathyroid hormone (PTH) was elevated (85.1 pg/ml). The patient was put on metoprolol 50 mg twice daily. The patient was also continued on intravenous (IV) hydration; electrolytes were supplemented. CT chest was done which confirmed the presence of old embolism and showed a 1.1 cm nodule in the left upper lobe (Figure 1).

Endocrinology was consulted for persistent tachycardia with relatively stable BP. The patient had spikes of temperature for two days and was empirically started on Tamiflu and Rocephin while waiting for blood culture. The patient was insisting on being discharged home when he suddenly collapsed and went into cardiorespiratory failure, then he was intubated and transferred to the intensive care unit (ICU) where attempts to resuscitate were futile. The family refused an autopsy. Result for pheochromocytoma workup received after the death of the patient and it showed elevated levels of 24-hour urine metanephrine at 2001 mcg and norepinephrine at 1499 mcg and Vanillylmandelic acid (VMA) at 6.6 mg/24 h.

## Discussion

Phaeochromocytomas are catecholamine-producing neuroendocrine tumors originating from chromaffin cells of the adrenal medulla or extra-adrenal paraganglia. Tumors from extra-adrenal chromaffin tissue are titled as extra-adrenal pheochromocytomas or paragangliomas. Our patient had pheochromocytoma although we do not have histopathology. Extra-adrenal tumors that may predominantly secrete epinephrine are rare in the organ of Zuckerkandl. In our patient, CT abdomen did not resolve any mass at the bifurcation of the aorta. It is likely that the pulmonary mass is paraganglioma. The stimulating effect of catecholamine explains patient tachycardia. The patient is normotensive due to beta 2 receptor stimulating effect of epinephrine secreted by tumor negating the alpha receptor stimulating effect of norepinephrine. Hypokalemia was partially associated with vomiting, but hypokalemia recurred after repletion of potassium and cessation of vomiting.

Increased epinephrine concentrations in the plasma secreted by pheochromocytoma induce hypokalemia through stimulation of beta 2 receptors causing activation of sodium potassium-ATPase in skeletal muscle and subsequent intracellular shift of potassium [8, 9]. Hypokalemia and potassium depletion is associated with decreased magnesium absorption within the loop and distal tubule that may lead to increased magnesium excretion [10]. Profound hypocalcemia requiring continuous intravenous calcium repletion was explained by the increased urinary loss of calcium due to hypomagnesemia and hypokalemia. Pheochromocytomas are known to secrete adrenomedullin. Its physiologic actions include increased Ca2 sequestration within the bones, leading to hypocalcemia. Adrenomedullin is similar to the calcitonin family and stimulates osteoblast activity and bone mineralization. Although, it has potent vasodilatory effects in several vascular systems, which may contribute to the hypotensive episodes [11]. Upon searching different literature database, no similar cases were found.

## Conclusions

The extra-adrenal tumor can also secrete enough epinephrine to negate the effect of norepinephrine leading to hypertension. The clinician should be aware of the possibility that tachycardia could be presenting symptom in pheochromocytoma despite the absence of hypertension especially if associated with hypokalemia and hypocalcemia.
